# Potential Clinical and Economic Value of Norovirus Vaccination in the Community Setting

**DOI:** 10.1016/j.amepre.2020.10.022

**Published:** 2021-01-27

**Authors:** Sarah M. Bartsch, Kelly J. O’Shea, Patrick T. Wedlock, Marie C. Ferguson, Sheryl S. Siegmund, Bruce Y. Lee

**Affiliations:** Public Health Informatics, Computational, and Operations Research (PHICOR), Graduate School of Public Health and Health Policy, City University of New York, New York City, New York

## Abstract

**Introduction:**

With norovirus vaccine candidates currently under development, now is the time to identify the vaccine characteristics and implementation thresholds at which vaccination becomes cost effective and cost saving in a community setting.

**Methods:**

In 2020, a norovirus transmission, clinical, and economics computational simulation model representing different U.S. population segments was developed to simulate the spread of norovirus and the potential impact of vaccinating children aged <5 years and older adults (aged ≥65 years).

**Results:**

Compared with no vaccination, vaccinating preschool-aged children averted 8%–72% of symptomatic norovirus cases in a community, whereas vaccinating older adults averted 2%–29% of symptomatic cases (varying with vaccine efficacy [25%–75%] and vaccination coverage [10%–80%]). Vaccination with a 25% vaccine efficacy was cost effective (incremental cost–effectiveness ratio ≤$50,000 per quality-adjusted life year) when vaccination cost ≤$445 and cost saving at ≤$370 when vaccinating preschool-aged children and ≤$42 and ≤$30, respectively, when vaccinating older adults. With a 50% vaccine efficacy, vaccination was cost effective when it cost ≤$1,190 and cost saving at ≤$930 when vaccinating preschool-aged children and ≤$110 and ≤$64, respectively, when vaccinating older adults. These cost thresholds (cost effective and cost saving, respectively) further increased with a 75% vaccine efficacy to ≤$1,600 and ≤$1,300 for preschool-aged children and ≤$165 and ≤$100 for older adults.

**Conclusions:**

This study outlines thresholds at which a norovirus vaccine would be cost effective and cost saving in the community when vaccinating children aged <5 years and older adults. Establishing these thresholds can help provide decision makers with targets to consider when developing and implementing a norovirus vaccine.

## INTRODUCTION

With multiple norovirus vaccine candidates under development,^1–3^ now is the time to identify the vaccine characteristics and implementation thresholds at which vaccination becomes cost effective and cost saving in a community setting. Establishing these thresholds can help provide decision makers (e.g., developers, funders, policymakers) with targets to consider when developing and implementing a norovirus vaccine. Children aged <5 years and older adults (aged ≥65 years) are likely to be initial target populations for vaccination, as these groups have the highest incidence of sporadic cases of norovirus in community settings (152.2 and 75.8 cases/1,000 people, respectively),^4^ where 90% of the annual economic burden occurs,^5^ and of norovirus-associated outpatient visits (25.6 and 7.8/1,000 people).^4^ Additionally, older adults experience higher rates of norovirus-associated hospitalizations (6.5–28.5/10,000 person years, varying with age) and deaths (18.8/1,000,000 person years), estimated from insurance claims,^6^ and generate higher costs ($751/illness).^5^ Although previous individual-based modeling work showed that vaccinating young children and older adults provided the most benefits (even generating cost savings), it did not account for potential reductions on transmission.^7^ A study including transmission showed that vaccinating children aged <5 years and older adults decreased cases in the total population but did not consider costs or measure health effects (e.g., quality-adjusted life years and disability-adjusted life years).^8^ Thus, there is a need to further explore and better understand the clinical and economic impact of a norovirus vaccine when accounting for transmission to guide decision making regarding vaccination implementation (e.g., target populations, coverage levels) and vaccine characteristics (e.g., efficacy and price points).

Currently, at least 3 vaccines are in Phase 1–2 clinical trials, and at least 3 others are in preclinical trials.^2^ To date, those in clinical trials have been found to be safe, well tolerated, and able to prompt an immune response and are undergoing further investigation.^9–11^ A challenge study found signs and symptoms of norovirus disease to be less common and less severe in vaccine recipients than controls.^9^ Evaluating a vaccine’s economic value before licensure can help guide development and implementation, efficacy profiles, and price points while there still is time to make adjustments.^12,13^ Therefore, this study uses a computational model to simulate norovirus spread and to evaluate the potential epidemiologic, clinical, and economic value of a norovirus vaccine in the population under varying circumstances.

## METHODS

In 2020, a norovirus transmission, clinical, and economics model was developed using Microsoft Excel with the Crystal Ball add-in representing different segments of the U.S. population to simulate norovirus spread and the potential impact of vaccination from the third-party payer and societal perspectives. The Appendix, available online, describes the model and its inputs, along with their values and data sources and model calibration.

### Norovirus Model Structure

Norovirus transmission was simulated with an age-structured compartment model consisting of 4 age groups (*x*): preschool-aged children (0–4 years), school-aged children (5–17 years), adults (18–64 years), and older adults (≥65 years). Appendix Figure 1, available online, outlines the model, which consisted of 7 mutually exclusive compartments: susceptible (S: not infected with norovirus but able to become infected), exposed (E: infected with norovirus but not yet able to transmit to others), infectious and symptomatic (Is: infected, experiencing symptoms, and able to transmit to others), infectious and asymptomatic post-symptoms (Ip: infected, no longer experiencing symptoms, but able to transmit to others), infectious and asymptomatic (Ia: infected, not experiencing symptoms, but able to transmit to others), recovered (R: immune and cannot become infected, either from vaccination or recovery from illness), and dead (D: leave the model).

Each person in the population, with an age following the U.S. age distribution, was represented in 1 of these compartments. All individuals in the model started in the S compartment. The model advanced in discrete, 1-day time steps through the course of 1 year, with individuals interacting with one another, based on age-specific contact patterns (Appendix, available online). On Day 1, a symptomatic norovirus infection was introduced into the population. Each day, the number of susceptible individuals in each age group (*x*) who became exposed (moving from the S to E compartment) was governed by the transmission coefficient (*β*) and number of individuals in the S, Is, Ip, and Ia compartments, according to the following equation:

$$\beta_{i}S_{X}Is_{a}+\beta_{j}S_{x}Is_{b}+\beta_{j}S_{X}Is_{c}+\beta_{j}S_{X}Is_{d}+\beta_{k}S_{x}\left(Ip_{a}+Ia_{a}\right)+\beta_{k}S_{x}\left(Ip_{a}+Ia_{b}\right)+\beta_{k}S_{x}\left(Ip_{a}+Ia_{c}\right)+\beta_{k}S_{x}\left(Ip_{a}+Ia_{d}\right),$$

where a–d represent different age groups (*x*) and *β* is age- and disease state–specific (represented by i–k; Appendix, available online).

Individuals remained in the E compartment for the incubation period duration before moving to 1 of the infectious compartments (at a rate of 1/incubation period duration). Individuals had a probability of being symptomatic, moving to the Is compartment, remaining in the Is compartment until their symptoms resolved, and moving to the Ip compartment (at a rate of 1/symptomatic illness duration). Those who did not experience symptoms moved directly into the Ia compartment. Individuals stayed in the Ia compartment for their viral shedding duration (i.e., infectious period duration), whereas individuals stayed in the Ip compartment for the remainder of their viral shedding duration after their symptoms resolve (i.e., total viral shedding duration minus symptomatic illness duration). After the viral shedding duration ended, individuals in the Ip and Ia compartments moved to the R compartment, remaining there for the duration of the simulation (although the duration and degree of natural immunity is not well understood, it is thought to last for at least a year^2,3,14^). Each day, any individual could die (i.e., all-cause mortality), moving to the D compartment. Additionally, those in the Is compartment could die from norovirus illness, moving to the D compartment at a rate equivalent to the probability of norovirus-associated mortality. Each day, new susceptible individuals entered the population in the group aged 0–4 years to maintain a stable population size (i.e., births equal deaths).

Vaccination was modeled to prevent disease (i.e., symptomatic illness), following a challenge study showing that an intramuscular vaccine reduced clinical symptoms^9^ and protected for a year (following natural immunity). Vaccinated individuals could become exposed and move to the E compartment; however, at the end of the incubation period, they had a lower probability of developing symptomatic infection (i.e., attenuated by vaccine efficacy). Thus, vaccinated individuals in the E compartment moved to the Ia compartment based on the probability of asymptomatic infection and vaccine efficacy, where they could actively transmit for the viral shedding duration before moving to the R compartment.

Each individual with symptomatic norovirus had probabilities of seeking medical care (i.e., outpatient or ambulatory care visits) and hospitalization. Additionally, each symptomatic individual had a probability of missing productive days (e.g., work or school) for a given duration. Those who were vaccinated had a probability of experiencing side effects (e.g., acute gastroenteritis), which were assumed to last the same duration as symptomatic norovirus illness.

The third-party payer perspective included all direct medical costs (i.e., vaccination, outpatient visits, and hospitalization), whereas the societal perspective included direct and indirect (i.e., productivity losses owing to absenteeism and mortality) costs. Daily wages served as a proxy for productivity losses. It was assumed that all symptomatic norovirus cases accrued productivity losses, regardless of age or employment status, as everyone is assumed to contribute to society. If a nonhospitalized case missed productive days, productivity losses accrued for the duration of their illness; if hospitalized, they accrued for their hospitalization duration. Mortality resulted in accruing the net present value of that person’s lifetime earnings, based on a person’s age of death and their remaining years of life based on life expectancy.^15,16^ All costs are in 2020 U.S. dollars, converted using a 3% discount rate.

For each scenario, the incremental cost–effectiveness ratio was calculated as:

$$({\text{Cost}}_{\text{Vaccination}}-{\text{Cost}}_{\text{NoVaccination}})\text{/}({\text{HealthEffects}}_{\text{NoVaccination}}-{\text{HealthEffects}}_{\text{Vaccination}}),$$

where health effects were measured by quality-adjusted life years (QALYs) and disability-adjusted life years (DALYs). Though QALYs are more commonly used in the U.S., DALYs allow for cross-country comparisons^17^; therefore, both are used in this study. QALYs are calculated as QALYs lost because of norovirus. Each norovirus illness losses QALYs based on their age-dependent QALY value attenuated by the gastroenteritis-specific utility weight for their illness duration. Death results in the loss of the net present value of QALYs for the remainder of the individual’s lifetime. DALYs are the sum of the years of life lived with disability and years of life lost because of norovirus-related deaths (Appendix, available online). Vaccination was considered cost effective when costing ≤$50,000 or ≤$100,000 per health effect gained (i.e., per QALY gained or DALY averted).

### Experiments and Sensitivity Analysis

Experiments consisted of Monte Carlo simulations of 2,000 trials, randomly drawing a value for each parameter from its distribution (Appendix Table 1, available online) at the beginning of each trial. Initial scenarios assumed no vaccination, whereas experimental scenarios consisted of vaccinating preschool-aged children and older adults, separately and in combination. Sensitivity analysis varied population size (2,500–7,500), vaccine cost ($1–$2,000), vaccine efficacy (25%–75%), and vaccination coverage (10%–80%). This study also determined the impact of vaccination on norovirus in the U.S.

## RESULTS

### No Vaccination

Without vaccination, in a population of 2,500 people, there was a median of 173 (95% uncertainty interval [UI]=111, 247) symptomatic norovirus cases (incidence: 69.4 per 1,000), 0.42 (95% UI=0.26, 0.64) QALYs lost, 0.72 (95% UI=0.44, 1.13) DALYs, 15 (95% UI=10, 21) ambulatory care visits, and 0.8 (95% UI=0.5, 1.1) hospitalizations, costing a median of $8,407 (95% UI=$5,050, $12,800) in direct medical costs and $81,510 (95% UI= $14,648, $218,140) in productivity losses ($89,917 total cost). The number of outcomes increased proportionally, doubling in a population of 5,000 (e.g., 350 norovirus cases, costing $17,268 in direct medical costs and $184,505 in productivity losses) and tripling in a population of 7,500 (e.g., 518 norovirus cases [incidence: 69.1 per 1,000], costing $24,756 in direct medical costs and $243,347 in productivity losses).

### Impact of Vaccinating Preschool-Aged Children (Aged 0–4 Years)

Vaccinating preschool-aged children reduced the burden of norovirus in the community, even with a 25% vaccine efficacy, resulting in a median of 162 (95% UI=103, 233) to 98 (95% UI=55, 178) symptomatic cases (10%–80% vaccination coverage, population: 2,500). Table 1 shows the mean number of clinical outcomes averted compared with no vaccination in the simulated population of 2,500; Table 2 shows the outcomes averted in the U.S. For example, with a 50% vaccine efficacy, vaccinating 10%–80% of preschool-aged children in the U.S. averted 3.8–14.9 million symptomatic cases, 0.3–1.3 million ambulatory care visits, and 15,851–64,725 hospitalizations.

**Figure 1.**
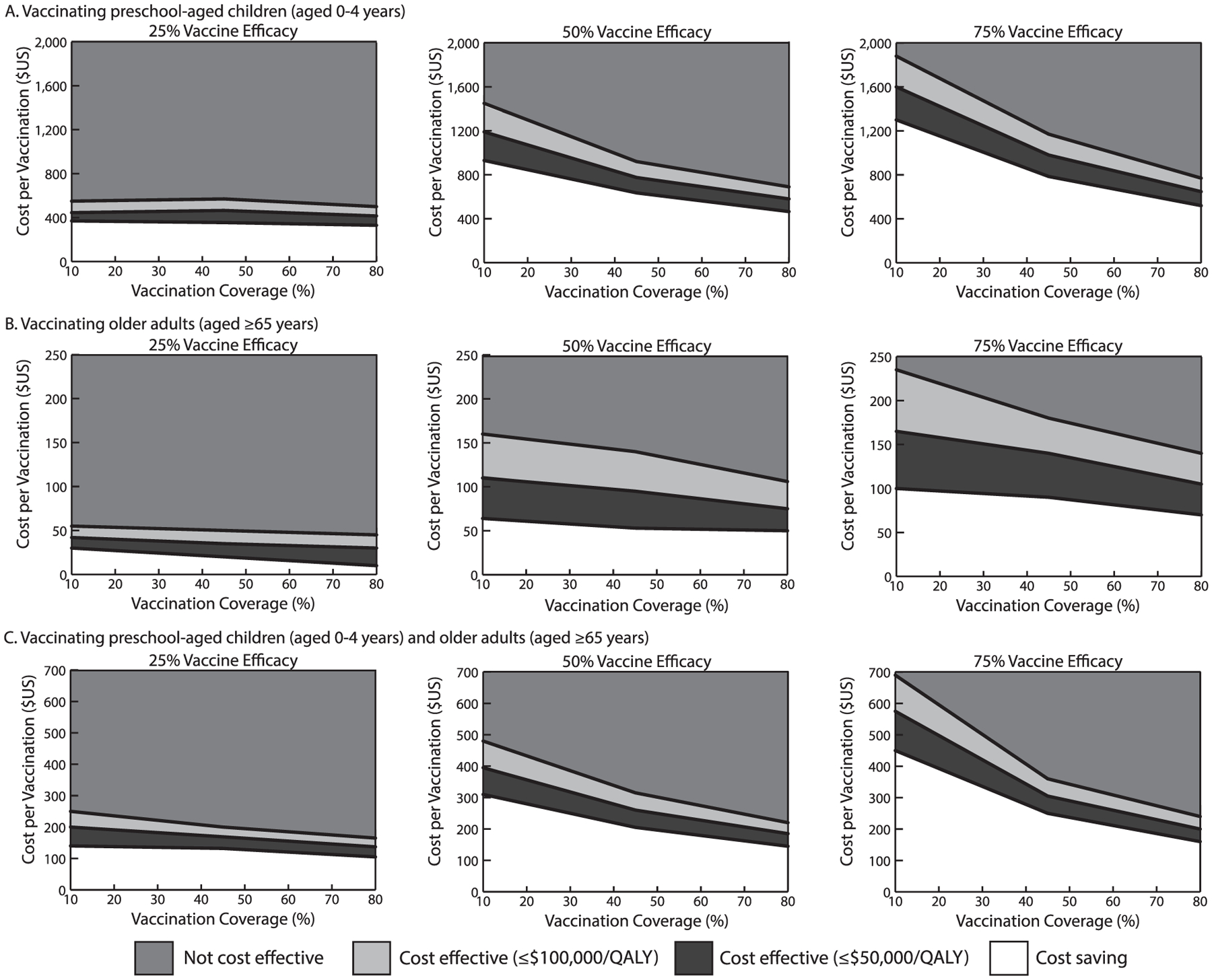
Vaccination cost and vaccination coverage at which n*orovirus* vaccination was cost effective^a^ compared with no vaccination across different vaccine efficacies from the societal perspective in a population of 2,500 persons when targeting (**A**) preschool-aged children (aged 0–4 years); (**B**) older adults (aged ≥65 years); and (**C**) preschool-aged children and older adults. *Notes:* Note difference in scales across panels. ^a^Incremental cost–effectiveness ratio ≤$50,000 and ≤$100,000 per QALY. QALY, quality-adjusted life year.

Figure 1 maps out the vaccination cost and coverage combinations at which vaccination was cost effective and cost saving (health effects measured in QALYs from the societal perspective) at different vaccine efficacies. For example, with a 10% vaccination coverage of preschool-aged children, vaccination must cost ≤$445 to be cost effective (≤$50,000/QALY) and ≤$370 to be cost saving (25% vaccine efficacy). Increasing vaccine efficacy to 50%, vaccination averted a median of ≥15 more symptomatic cases than a 25% vaccine efficacy (coverages ≥10%) and increased the cost thresholds (Figure 1A). For example, for vaccination to be cost effective (≤$50,000/QALY) when coverage was 10%, vaccination must cost ≤$1,190, and with a 45% vaccination coverage, vaccination must cost ≤$635 to be cost saving. When vaccine efficacy was further increased to 75%, vaccination averted a median of ≥11 more symptomatic cases than a 50% vaccine efficacy (coverages ≥10%), increasing the cost threshold at which vaccination was cost effective and cost saving (Figure 1A; e.g., ≤$1,300 to be cost saving with a 10% coverage).

**Figure 2.**
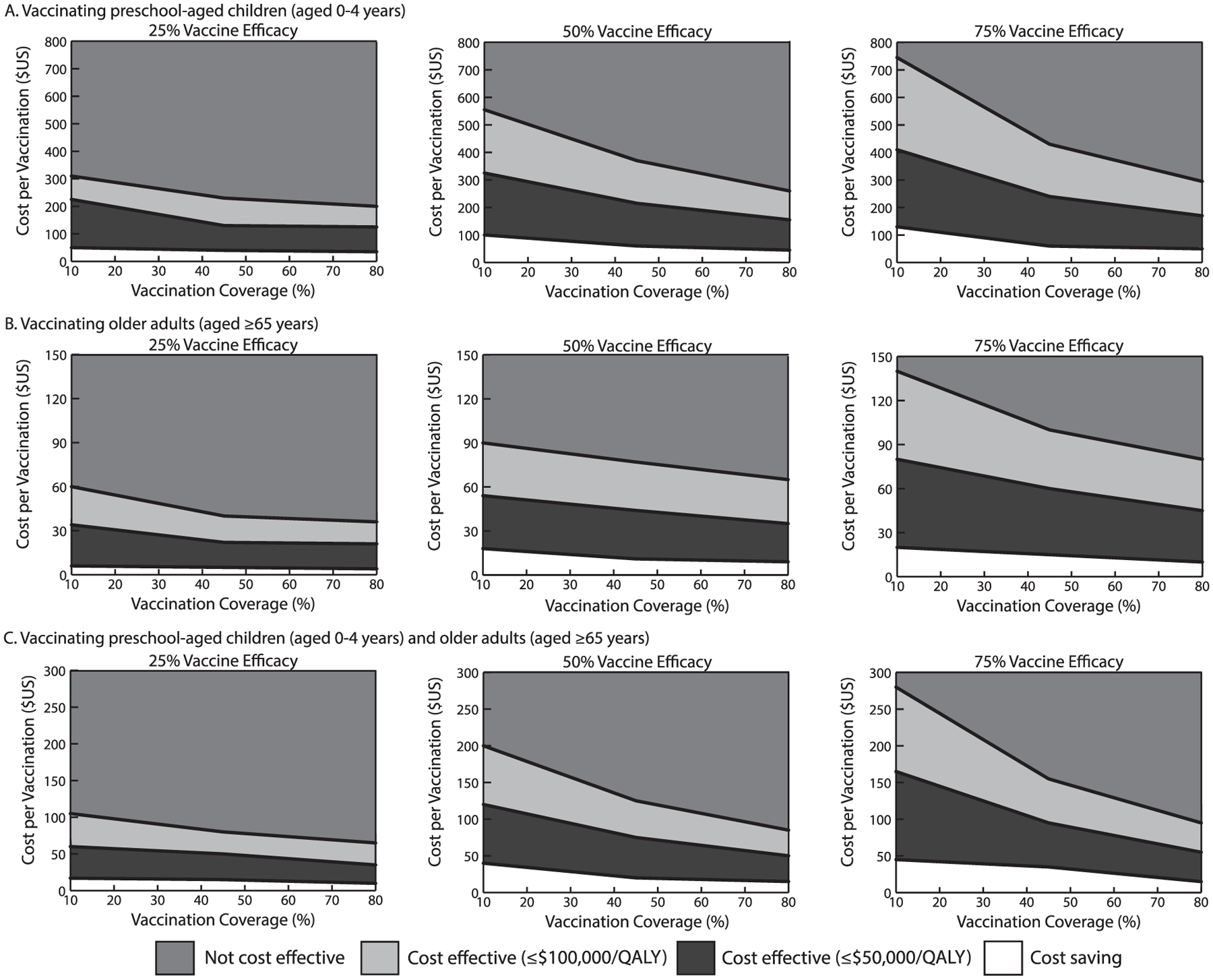
Vaccination cost and vaccination coverage at which n*orovirus* vaccination was cost effective^a^ compared with no vaccination across different vaccine efficacies from the third-party payer perspective in a population of 2,500 persons when targeting (**A**) preschool-aged children (aged 0–4 years); (**B**) older adults (aged ≥65 years); and (**C**) preschool-aged children and older adults. *Notes:* Note difference in scales across panels. ^a^Incremental cost–effectiveness ratio ≤$50,000 and ≤$100,000 per QALY. QALY, quality-adjusted life year.

As Figure 2 shows, thresholds were substantially lower from the third-party payer perspective, as the vast majority of savings from averted cases stem from reduced productivity losses. For example, with a 10% coverage, it must cost ≤$325 to be cost effective and ≤$90 to be cost saving with a 50% vaccine efficacy.

When measuring health effects in DALYs (Appendix Figure 2, available online), the cost thresholds at which vaccination was cost effective and cost saving were higher than when measuring QALYs. For example, with a 10% coverage of preschool-aged children, vaccination must cost ≤$665 to be cost effective and ≤$480 to be cost saving with a 25% vaccine efficacy and ≤$1,360 and ≤$650 with a 50% vaccine efficacy.

Clinical outcomes and their associated costs increased proportionally with population size; for example, with a 50% vaccine efficacy, vaccination resulted in 145 (95% UI=95, 211) cases in a population of 2,500 and 430 (95% UI=282, 629) cases in a population of 7,500 (10% vaccination coverage). However, cost effective and cost saving thresholds held with changes in population size (Appendix Figure 3A, available online).

### Impact of Vaccinating Older Adults (Aged ≥65 Years)

With a 25% vaccine efficacy, vaccinating older adults resulted in 170 (95% UI=110, 241) to 151 (95% UI=96, 224) symptomatic cases (10%–80% vaccination coverage, population: 2,500). Compared with no vaccination, vaccination significantly reduced the mean number of clinical outcomes (Tables 1 and 2). Figure 1B maps out the vaccination cost and coverage combinations that were cost effective and cost saving (cost/QALY). For example, with a 25% vaccine efficacy and 10% coverage of older adults, vaccination must cost ≤$42 to be cost effective and ≤$30 to be cost saving. Increasing vaccine efficacy further increases these cost thresholds (Figure 1B); for example, with a 45% coverage of older adults, vaccination must cost ≤$95 to be cost effective and ≤$53 to be cost saving with a 50% vaccine efficacy and ≤$140 and ≤$90 with a 75% vaccine efficacy. Thresholds were substantially lower from the third-party payer perspective (Figure 2B); for example, with a 10% coverage of older adults, it must cost ≤$80 to be cost effective with a 75% vaccine efficacy.

When measuring health effects in DALYs (Appendix Figure 2B, available online), cost thresholds were generally higher. For example, with a 10% vaccination coverage and 75% vaccine efficacy, it must cost ≤$275 to be cost effective and ≤$125 to be cost saving.

When increasing population size, the number of clinical outcomes increased proportionally (e.g., with a 50% vaccine efficacy, vaccination resulted in 168 [95% UI=108, 237] symptomatic cases in a population of 2,500 and 444 [95% UI=320, 710] in a population of 7,500 [10% vaccination coverage of older adults]), whereas the vaccination cost and vaccine efficacy thresholds were relatively stable (Appendix Figure 3B, available online).

### Impact of Vaccinating Preschool-Aged Children and Older Adults

Vaccinating both preschool-aged children and older adults averted more cases than vaccinating either population alone. For example, with a 50% vaccine efficacy, vaccinating both populations (55 people/2,500 population) averted a median of 35–123 cases, 88–304 missed productive days, and 3–11 ambulatory care visits compared with no vaccination (10%–80% vaccination coverage). As Figures 1C and 2C show, the combinations of vaccination cost and coverage that would be cost effective and cost saving fell between vaccinating either age group alone. With a 75% vaccine efficacy (10% coverage), vaccination was cost effective at ≤$575 and cost saving at ≤$450.

## DISCUSSION

This study outlines vaccine characteristic and vaccination implementation thresholds at which a norovirus vaccine would be cost effective and cost saving in the community when vaccinating children aged <5 years and older adults. Even with a 25% vaccine efficacy and 10% vaccination coverage, a norovirus vaccine could decrease symptomatic cases in a community by a relative 7.7%, saving at least $8,000 in norovirus-associated direct medical costs and productivity losses. Vaccinating children aged <5 years garnered the largest benefits in the total population, with a relative ≤72% decrease in symptomatic cases. As children aged <5 years contribute substantially to norovirus’ spread, vaccinating just 10% of preschool-aged children impacts spread and subsequent norovirus-associated costs. Although vaccinating older adults provided total population benefits (≤28.5% relative reduction), these reductions were modest compared with vaccinating preschool-aged children. These findings generally hold when varying population size. This study builds further evidence of a norovirus vaccine’s value^7,8^ by accounting for the economic and clinical value in the context of transmission in different population sizes.

This study found that, when including transmission, the vaccination cost could be as high as $1,300 and still provide cost savings and as high as $1,600 and still be cost effective. Cost thresholds were substantially lower from the third-party payer perspective (i.e., ≤$130 to still provide cost savings and ≤$410 to still be cost effective). This is not surprising given that productivity losses represent 76.4%–93.6% of norovirus’ cost per case.^5^ Additionally, cost thresholds vary with vaccination age, as these thresholds depend on factors such as the cost per case, norovirus risk, and how much they transmit in the community. For example, thresholds were higher for preschool-aged children given their substantial role in transmission. It should be noted that this cost includes the vaccine, administration, and other associated costs. Pricing a vaccine can be challenging,^12,18^ as showing that a higher price can be supported may support development; however, the vaccine also needs to be affordable to be of broader use.

These results show that the vaccination coverage only needs to be 10% to provide clinical and economic benefits in the community. This coverage could be achieved in a few ways, such as vaccination before daycare attendance or part of routine immunizations. Although this coverage does not seem high, a norovirus vaccine would likely need to be given yearly (annual vaccination), posing challenges, and achieving coverage may be difficult, as is seen with yearly influenza vaccination. Effectively reaching older adults could also be challenging, given gaps in coverage and barriers to access (e.g., lack of regular wellness visits, lack of reminders) for many recommended vaccines.^19,20^ This study also found that the cost thresholds varied with increasing vaccination coverage as there is a trade-off between the additional reduction in cases and increased vaccination costs.

This study aimed to be conservative about the vaccination’s value. For example, it only considered a vaccine that prevents disease; a vaccine preventing infection would reduce transmission, increasing the vaccine’s value. Additionally, vaccination may also reduce the chances of an outbreak or mitigate spread (e.g., in daycare or long-term care settings), further increasing the vaccination’s value. Additionally, potential costs that may be incurred after hospitalization during recovery (e.g., home healthcare or productivity losses) were not included.

### Limitations

All models, by definition, are simplifications of real life and cannot account for every possible factor or outcome.^21^ Although the model is calibrated for effective population sizes that represented transmission patterns reported in the literature, norovirus spread in the community may not conform to the model data. Model inputs drew from various sources and results may change as new data emerge. POLYMOD social mixing data may not be representative of contacts for norovirus. Additionally, genetic factors that may make some individuals resistant to infection were not modeled directly.

## CONCLUSIONS

This study outlines thresholds at which a norovirus vaccine would be cost effective (e.g., ≤$1,600 and ≤$410 to society and third-party payers, respectively) and cost saving (e.g., ≤$1,300 and ≤$130) in the community.

## Supplementary Material

Appendix

## Figures and Tables

**Table 1.** Number of Clinical Outcomes Averted With *Norovirus* Vaccination Compared With No Vaccination (2,500-Person Population)

Vaccination scenario	Total norovirus cases averted Mean (95% CI)	Symptomatic cases averted Mean (95% CI)	Deaths averted Mean (95% CI)	Missed productive days averted Mean (95% CI)	Ambulatory care visits averted Mean (95% CI)	Hospitalizations averted Mean (95% CI)
Vaccinating preschool-aged children (aged 0–4 years)						
25% vaccine efficacy						
10% coverage	19.8 (19.5, 20.1)	13.6 (13.4, 13.8)	0.01 (0.01, 0.01)	37.1 (36.7, 37.5)	1.2 (1.1, 1.3)	0.06 (0.04, 0.08)
45% coverage	79.4 (79.1, 79.7)	54.2 (54.0, 54.4)	0.01 (0.01, 0.01)	140.5 (140.1, 140.8)	4.8 (4.7, 4.8)	0.24 (0.22, 0.26)
80% coverage	113.7 (113.5, 114.0)	77.6 (77.4, 77.8)	0.02 (0.02, 0.02)	201.1 (200.8, 201.5)	6.8 (6.8, 6.9)	0.34 (0.32, 0.36)
50% vaccine efficacy						
10% coverage	41.7 (41.4, 42.0)	28.5 (28.3, 28.7)	0.01 (0.01, 0.01)	73.3 (73.0, 73.7)	2.5 (2.4, 2.6)	0.12 (0.11, 0.13)
45% coverage	130.0 (129.7, 130.2)	88.3 (88.1, 88.5)	0.02 (0.02, 0.02)	223.0 (222.7, 223.3)	7.8 (7.7, 7.8)	0.38 (0.37, 0.39)
80% coverage	166.4 (166.1, 166.6)	113.0 (112.8, 113.2)	0.02 (0.02, 0.02)	284.8 (284.5, 285.1)	9.9 (9.8, 9.9)	0.49 (0.48, 0.50)
75% vaccine efficacy						
10% coverage	59.1 (58.8, 59.4)	40.3 (40.1, 40.5)	0.01 (0.01, 0.01)	101.6 (101.2, 101.9)	3.6 (3.5, 3.6)	0.18 (0.17, 0.19)
45% coverage	158.7 (158.5, 159.0)	107.7 (107.5, 107.9)	0.02 (0.02, 0.02)	270.8 (270.5, 271.1)	9.4 (9.4, 9.5)	0.47 (0.46, 0.48)
80% coverage	186.3 (186.1, 186.5)	126.5 (126.3, 126.6)	0.02 (0.02, 0.02)	316.6 (316.3, 316.8)	11.1 (11.0, 11.1)	0.55 (0.54, 0.56)
Vaccinating older adults (aged ≥65 years)						
25% vaccine efficacy						
10% coverage	3.9 (3.6, 4.1)	3.3 (3.0, 3.5)	0.01 (0.01, 0.01)	8.1 (7.7, 8.5)	0.3 (0.2, 0.4)	0.03 (0.01, 0.05)
45% coverage	15.9 (15.6, 16.2)	13.5 (13.2, 13.7)	0.01 (0.01, 0.01)	36.2 (35.8, 36.6)	1.2 (1.1, 1.2)	0.10 (0.08, 0.12)
80% coverage	25.1 (24.8, 25.4)	21.5 (21.3, 21.8)	0.01 (0.01, 0.01)	56.0 (55.6, 56.4)	1.9 (1.8, 1.9)	0.16 (0.15, 0.17)
50% vaccine efficacy						
10% coverage	7.0 (6.7, 7.3)	6.0 (5.8, 6.3)	0.01 (0.01, 0.01)	16.1 (15.8, 16.5)	0.5 (0.4, 0.6)	0.05 (0.03, 0.07)
45% coverage	30.7 (30.4, 30.9)	25.6 (25.4, 25.8)	0.01 (0.01, 0.01)	62.4 (62.1, 62.8)	2.2 (2.1, 2.3)	0.19 (0.18, 0.20)
80% coverage	45.9 (45.6, 46.2)	38.5 (38.3, 38.7)	0.02 (0.02, 0.02)	96.5 (96.1, 96.9)	3.3 (3.3, 3.4)	0.28 (0.27, 0.29)
75% vaccine efficacy						
10% coverage	11.5 (11.2, 11.8)	9.7 (9.4, 9.9)	0.01 (0.01, 0.01)	23.8 (23.4, 24.2)	0.8 (0.8, 0.9)	0.07 (0.05, 0.09)
45% coverage	42.0 (41.7, 42.3)	35.0 (34.7, 35.2)	0.02 (0.02, 0.02)	88.1 (87.8, 88.5)	3.0 (3.0, 3.1)	0.25 (0.24, 0.26)
80% coverage	59.9 (59.6, 60.2)	50.1 (49.9, 50.3)	0.02 (0.02, 0.02)	124.6 (124.2, 124.9)	4.3 (4.2, 4.4)	0.37 (0.36, 0.38)

*Note:* When vaccinating preschool-aged children: 10% coverage=15, 45% coverage=68, 80% coverage=121; when vaccinating older adults: 10% coverage=40, 45% coverage=180, 80% coverage=320.

**Table 2.** Mean Number of Clinical Outcomes Averted in the United States With *Norovirus* Vaccination Compared With No Vaccination

Vaccination scenario	Total norovirus cases averted Mean	Symptomatic cases averted Mean	Deaths averted Mean	Missed productive days averted Mean	Ambulatory care visits averted Mean	Hospitalizations averted Mean
Vaccinating preschool-aged children (aged 0–4 years)						
25% vaccine efficacy						
10% coverage	2,615,414	1,796,446	1,321	4,900,598	158,510	7,925
45% coverage	10,488,073	7,159,365	1,321	18,558,870	634,040	31,702
80% coverage	15,018,815	10,250,308	2,642	26,563,621	898,223	44,911
50% vaccine efficacy						
10% coverage	5,508,220	3,764,611	1,321	9,682,314	330,229	15,851
45% coverage	17,171,908	11,663,688	2,642	29,456,427	1,030,314	50,195
80% coverage	21,980,042	14,926,351	2,642	37,619,688	1,307,707	64,725
75% vaccine efficacy						
10% coverage	7,806,614	5,323,291	1,321	13,420,507	475,530	23,776
45% coverage	20,962,937	14,226,265	2,642	35,770,405	1,241,661	62,083
80% coverage	24,608,665	16,709,587	2,642	41,820,201	1,466,217	72,650
Vaccinating older adults (aged ≥65 years)						
25% vaccine efficacy						
10% coverage	515,157	435,902	1,321	1,069,942	39,627	3,963
45% coverage	2,100,256	1,783,237	1,321	4,781,716	158,510	13,209
80% coverage	3,315,499	2,839,969	1,321	7,397,130	250,974	21,135
50% vaccine efficacy						
10% coverage	924,641	792,550	1,321	2,126,675	66,046	6,605
45% coverage	4,055,212	3,381,545	1,321	8,242,516	290,602	25,097
80% coverage	6,063,004	5,085,527	2,642	12,746,839	435,902	36,986
75% vaccine efficacy						
10% coverage	1,519,053	1,281,289	1,321	3,143,780	105,673	9,246
45% coverage	5,547,847	4,623,206	2,642	11,637,270	396,275	33,023
80% coverage	7,912,287	6,617,789	2,642	16,458,613	567,994	48,874

*Note:* 2020 U.S. population: 330,229,000.
