# Supplementary material for: Potential Clinical and Economic Value of Norovirus Vaccination in the Community Setting
Source: Am J Prev Med. Author manuscript; Available in PMC 2021 Sep 3. (PMC8415104; doi:10.1016/j.amepre.2020.10.022)
Supplement: Appendix [file NIHMS1720018-supplement-Appendix.docx]

Appendix to: *Potential Clinical and Economic Value of Norovirus Vaccination in the Community Setting*

Appendix Figure 1. Norovirus transmission, clinical, and economic outcomes model.


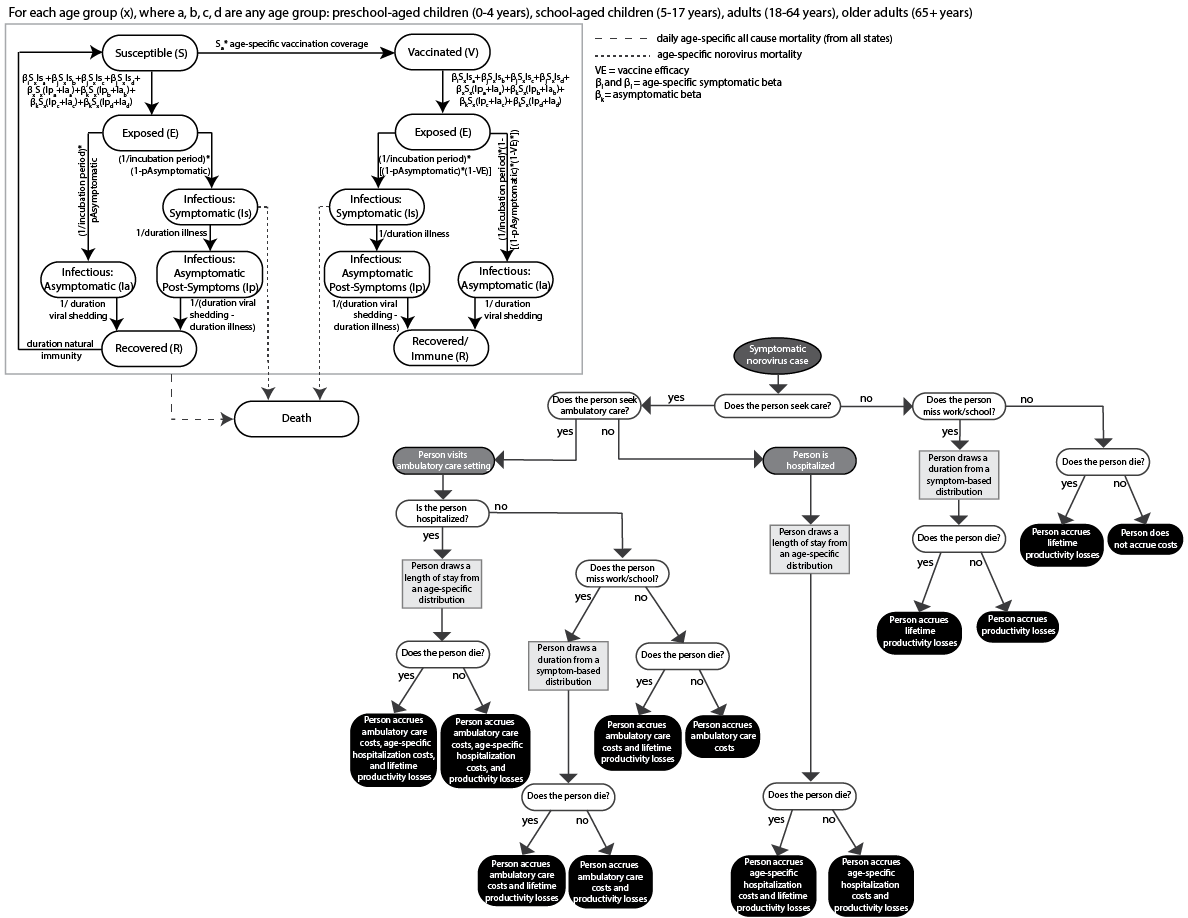


*Additional Norovirus Model Details*

Beta is a function of the reproduction number (R_0_, the average number of secondary cases generated by one infectious case), the duration of infectiousness, the probability that a given contact is among those in a specific age group, and the population size of the infectious contact’s age group each day. Based on the literature, we assumed different R_0_ values for symptomatic and asymptomatic individuals, and different values for those 0-4 years and ≥5 years. Additionally, we assumed symptomatic individuals were more infectious when experiencing symptoms than after symptoms resolve, since asymptomatically infected individuals contribute less to transmission than symptomatic individuals.^1^ To do this, we weighted R_0_ so that 95% of its value was incurred during the duration of symptoms, thereby accounting for the lower rate of viral shedding when asymptomatic.^2^

Health effects were measured by quality-adjusted life years (QALYs) and disability-adjusted life years (DALYs). DALYs (which allow for cross-country comparisons^3^) are the sum of the years of life lived with disability (YLD) and years of life lost (YLL) due to norovirus-related deaths. YLL and YLD are calculated as:

*YLD=Number of Incident Cases x Disability Weight x Average Duration in Years*

*YLL=Number of Deaths x Life Expectancy at Age of Death in Years*

*Data Sources*

Appendix Table 1 summarizes key model input parameters, values, and sources. All costs, clinical probabilities, and durations were age-specific when available and come from the scientific literature or nationally representative data sources. We derived the age-specific probability of hospitalization by dividing US estimates of norovirus hospitalizations^4^ by US norovirus incidence,^5, 6^ as described in our previous publication.^7^ In the absence of norovirus-specific utility weights, we used weights for infectious gastroenteritis as a proxy for norovirus. Disability weights came from the 2017 Global Burden of Disease^8^; non-hospitalized cases use weights for moderate diarrheal disease, while hospitalized cases used severe diarrheal disease. In the absence of data to characterize contacts and mixing among individuals between the various age groups in the US, we used data from the POLYMOD dataset.^9^ POLYMOD data comes from a contact survey conducted with daily diaries detailing the number of contacts, their age, duration, and whether they were physical, in eight different European countries.^10^ While these data may be more appropriate for pathogens spread through the air (e.g., influenza) as they include conversational contacts, we used this data as a proxy, as have previous models of pathogens transmitted via the fecal-oral route, including both rotavirus and norovirus.^11-14^ First, we determined the proportion of contacts per day for each age-group. To determine the proportions of contacts between each age group (0-4, 5-17, 18-64, and 65 and up), we added up the number of contacts reported in POLYMOD between each age group and then transformed it into a rate by dividing by the number of participants in each age group. Next, we corrected the reported rates for asymmetries in reporting by applying the equation described by Eames et al.^15^ We converted the corrected rates into proportions by dividing each rate by the total of all rates reported by each respective age group.

*Model Calibration*

We populated our model with three different populations and calibrated each to generate the trends of norovirus in the absence of vaccination. Specifically, we targeted generating the current U.S. estimate for the total population incidence of norovirus as reported by Grytdal et al. (68.9 per 1,000 person-years)^16^, as well as matching general trends in the age-specific incidence, such that adults 18 to 44 years had the largest number of cases and 0-4 year olds had the highest incidence per 1,000 persons. To do this, we attenuated the probability of contacts by age to obtain similar trends in the age distribution of cases seen in epidemiologic data, thus accounting for factors such as susceptibility to norovirus (e.g., accounting for genetic factors such as non-secretors who may be resistant to GII.4 norovirus infection^17^), and behaviors that may reduce transmission (e.g., sanitation and hygiene, etc.). For a population of 2,500, this calibration resulted in an overall population incidence of 68.9 per 1,000 persons with an incidence of 147.5 cases per 1,000 among 0-4 year olds (13.0% of cases); 25.6 cases per 1,000 among 5-17 year olds (6.1% of cases) 71.8 cases per 1,000 among 18-64 year olds (64.1% of cases); and 72.5 cases per 1,000 among those 65 years (16.8% of cases). This age-breakdown followed that reported by Grytdal et al.

After calibrating to these general trends, we varied the seeding in the population to target the total population incidence. These newly introduced infections represent reintroducing norovirus into the population by ways such as an infected person entering the population, environmental transmission, foodborne transmission, etc. For a population of 5,000 persons, this was equivalent to seeing a new case among older adults (65 years and older) every 21 days. This parameterization yielded a total incidence of 68.9 per 1,000, with the following age breakdown: 143.2 cases per 1,000 among 0-4 year olds (12.6% of total cases) 22.4 cases per 1,000 among 5-17 year olds (5.3% of total cases); 70.9 per 1,000 among 18-64 year olds (63.3% of total cases), and 80.7 per 1,000 among those 65 years and older (18.8% of total cases). For 7,500 persons, it was equivalent to seeing a new case among preschool-aged children every 60 days. This parameterization yielded an overall incidence of 68.7 per 1,000 persons, with the following age breakdown: 167.5 cases per 1,000 among 0-4 year olds (14.8% of total cases; 22.7 cases per 1,000 among 5-17 year olds (5.4% of total cases); 71 per 1,000 among 18-64 year olds (63.6% of total cases); and 69.4 per 1,000 among those 65 years (16.2% of total cases). While the age of the additional seed needed for each population size varied, they were found generate the best estimate of the overall population incidence while maintaining the age-specific trends.

Appendix Table 1. Model input parameters, values, and sources.

| **Parameter** | **Mean or Median** | **Standard Deviation or Range** | **Source** |
| --- | --- | --- | --- |
| ***Costs ($US, 2020)*** |  |  |  |
| Annual wage | 55,124.36 | 21,950.02 - 104,403.17 | ^18^ |
| Ambulatory care | 142.29 | 32.25 - 308.13 | ^19^ |
| Hospitalization |  |  |  |
| 0 to 17 years | 5,948.38 | 1,530.66 | ^20^ |
| 18 to 44 years | 5,684.51 | 426.97 | ^20^ |
| 45 to 64 years | 6,817.85 | 134.70 | ^20^ |
| 65 to 84 years | 8,097.97 | 543.44 | ^20^ |
| 85 years and older | 8,204.50 | 202.47 | ^20^ |
| ***Probabilities*** |  |  |  |
| Vaccination side effects | 0.098 | 0.011 - 0.14 |  |
| Probability of asymptomatic infection | 0.33 |  | ^21, 22^ |
| Relative infectiousness during asymptomatic period | 0.05 | 0.0432 - 0.0528* | ^2^ |
| Missing productive days (school or work) | 1 |  | Assumption |
| Seeking ambulatory care^ |  |  |  |
| 0 to 15 years | 0.168 | 0.1512 - 0.1848 | ^23^ |
| 16 to 25 years | 0.062 | 0.0558 - 0.062 | ^23^ |
| 26 to 45 years | 0.064 | 0.0576 - 0.0704 | ^23^ |
| 46 to 65 years | 0.054 | 0.0486 - 0.0594 | ^23^ |
| 65 years and older | 0.103 | 0.0927 - 0.1133 | ^23^ |
| Hospitalization |  |  |  |
| 0 to 4 years | 0.004363 | 0.0039 - 0.0048 | ^4-6^ |
| 5 to 17 years | 0.001836 | 0.0017 - 0.0020 | ^4-6^ |
| 18 to 64 years | 0.002194 | 0.0020 - 0.0024 | ^4-6^ |
| 65 to 74 years | 0.006916 | 0.0062 - 0.0076 | ^4-6^ |
| 75 to 84 years | 0.018288 | 0.0165 - 0.0201 | ^4-6^ |
| 85 years and older | 0.031391 | 0.02825 - 0.0345 | ^4-6^ |
| Norovirus-associated mortality |  |  |  |
| 0 to 4 years | 0.000006369 | 0.0000057 - 0.0000070 | ^6, 24^ |
| 5 to 64 years | 0.000004546 | 0.0000041 - 0.000005 | ^6, 24^ |
| 65 years and older | 0.000334003 | 0.000301 - 0.000367 | ^6, 24^ |
| ***Durations (in days)*** |  |  |  |
| Incubation period | 1.5 | 1 - 2 | ^25-27^ |
| Viral shedding (infectious period) | 15 | 5 - 34 | ^28-32^ |
| Illness |  | 2 - 3 | ^25-27^ |
| Natural immunity | ≥365 |  | ^33-35^ |
| Hospitalization |  |  |  |
| 0 to 17 years | 2.43 | 0.16 | ^20^ |
| 18 to 44 years | 2.76 | 0.06 | ^20^ |
| 45 to 64 years | 3.33 | 0.10 | ^20^ |
| 65 to 84 years | 3.60 | 0.07 | ^20^ |
| 85 years and older | 4.18 | 0.15 | ^20^ |
| ***Numbers*** |  |  |  |
| Reproductive rate (R_0_) |  |  |  |
| 0 to 4 years | 3.4 | 3.06 - 3.74* | ^36, 37^ |
| 5 years and up | 1.5 | 1.35 - 1.65* | ^1, 36, 38^ |
| Asymptomatic | 0.85 | 0.765 - 0.935* | ^1^ |
| ***Disability Weights*** |  |  |  |
| Moderate diarrheal disease^ | 0.188 | 0.125 - 0.264 | ^8^ |
| Severe diarrheal disease^◊^ | 0.247 | 0.164 - 0.348 | ^8^ |
| ***Utility Weights*** |  |  |  |
| Healthy QALY |  |  |  |
| 0 to 17 years | 1 |  | ^39^ |
| 18 to 64 years | 0.92 |  | ^39^ |
| 65 years and older | 0.84 |  | ^39^ |
| Mild/moderate gastroenteritis^ | 0.878 | 0.084 | ^40-49^ |
| Severe gastroenteritis^◊^ | 0.729 | 0.139 | ^40-45^ |

*Values are +/-10% of median value

^Weight for non-hospitalized norovirus cases

^◊^Weight for hospitalized norovirus cases

Appendix Figure 2. Vaccination cost and vaccination coverage at which norovirus vaccination was cost-effective [incremental cost-effectiveness ratio ≤$50,000 and ≤$100,000 / disability-adjusted life year (DALY) averted] compared to no vaccination across different vaccine efficacies from the societal perspective in a population of 2,500 persons when targeting A) preschool-aged children (0-4 years old); B) older adults (65 years and older); and C) preschool-aged children and older adults. Note difference in scales across panels.


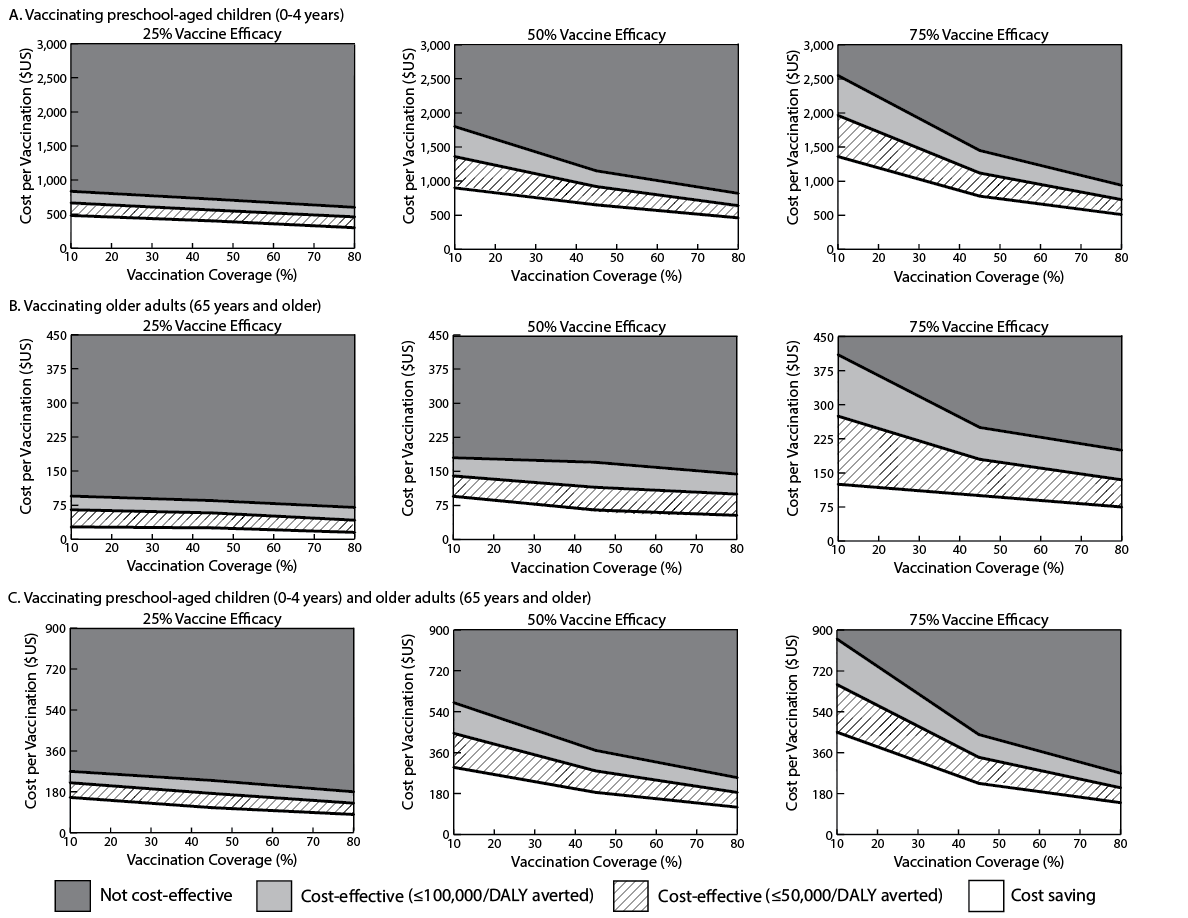


Figure 3. Vaccination cost and vaccination coverage at which norovirus vaccination was cost-effective [incremental cost-effectiveness ratio ≤$50,000 and ≤$100,000 per quality-adjusted life year (QALY) and per disability-adjusted life year (DALY) averted] compared to no vaccination with a vaccine efficacy of 50% from the societal perspective in a population of 7,500 persons when targeting A) preschool-aged children (0-4 years old); B) older adults (65 years and older); and C) preschool-aged children and older adults. Note difference in scales across panels.

**
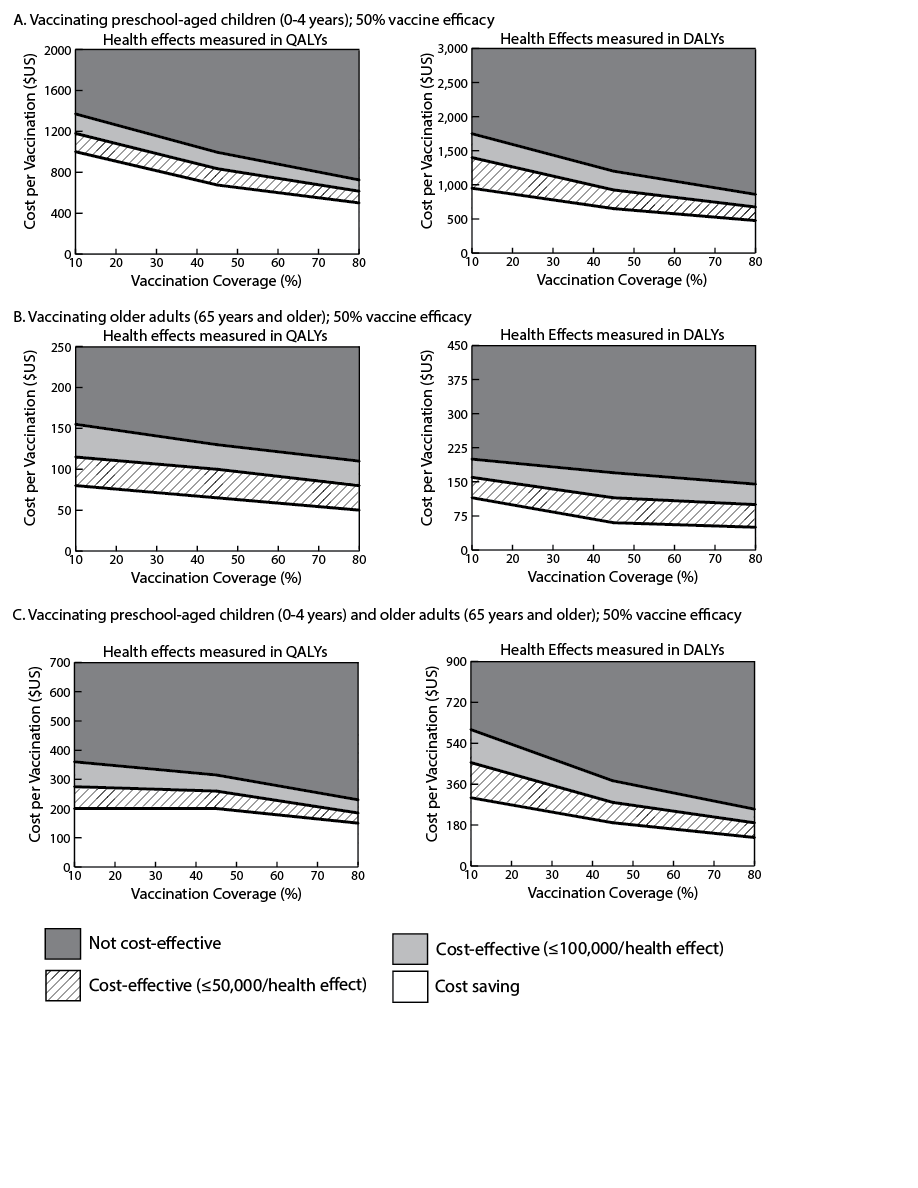
**

**References**

1. Sukhrie FH, Teunis P, Vennema H, Copra C, Beersma MFT, Bogerman J, et al. Nosocomial transmission of norovirus is mainly caused by symptomatic cases. Clinical Infectious Diseases 2012;54(7):931-937.

2. Atmar RL, Opekun AR, Gilger MA, Estes MK, Crawford SE, Neill FH, et al. Norwalk virus shedding after experimental human infection. Emerging Infectious Diseases 2008;14(10):1553-7.

3. Lee BY, Bartsch SM, Gorham KM. Economic and financial evaluation of neglected tropical diseases. Advances in Parasitology 2015;87:329-417.

4. Lopman BA, Hall AJ, Curns AT, Parashar UD. Increasing rates of gastroenteritis hospital discharges in US adults and the contribution of norovirus, 1996-2007. Clinical Infectious Diseases 2011;52(4):466-474.

5. Phillips G, Tam CC, Conti S, Rodriguez LC, Brow D, Iturriza-Gomara M, et al. Community incidence of norovirus-associated infectious intestinal disease in England: improved estimates using viral load for norovirus diagnosis. American Journal of Epidemiology 2010;171(9):1014-1022.

6. US Census Bureau, Population Division. Annual Estimates of the Resident Population for Selected Age Groups by Sex for the United States, States, Counties and Puerto Rico Commonwealth and Municipios: April 1, 2010 to July 1, 2018. 2019 June 2019 [cited 2019 October 17]; Available from: <https://factfinder.census.gov/faces/tableservices/jsf/pages/productview.xhtml?pid=PEP_2018_PEPAGESEX&prodType=table>

7. Bartsch SM, Lopman BA, Hall AJ, Parashar UD, Lee BY. The potential economic value of a human norovirus vaccine for the United States. Vaccine 2012;30(49):7097-104.

8. Global Burden of Disease Collaborative Network. Global Burden of Disease Study 2017 (GBD 2017) Disability Weights. In. Seattle, Washington Institute for Health Metrics and Evaluation (IHME); 2018.

9. Mossong J, Hens N, Jit M, Beutels P, Auranen K, Mikolajczyk R, et al. POLYMOD social contact data (Version 1.1) 2017 [cited 2019 November 20]; Available from: <http://doi.org/10.5281/zenodo.1215899>

10. Mossong J, Hens N, Jit M, Beutels P, Auranen K, Mikolajczyk R, et al. Social contacts and mixing patterns relevant to the spread of infectious diseases. PLoS Med 2008;5(3):e74.

11. Atchison C, Lopman B, Edmunds WJ. Modelling the seasonality of rotavirus disease and the impact of vaccination in England and Wales. Vaccine 2010;28(18):3118-26.

12. Bilcke J, Chapman R, Atchison C, Cromer D, Johnson H, Willem L, et al. Quantifying Parameter and Structural Uncertainty of Dynamic Disease Transmission Models Using MCMC: An Application to Rotavirus Vaccination in England and Wales. Med Decis Making 2015;35(5):633-47.

13. Van Effelterre T, Soriano-Gabarro M, Debrus S, Claire Newbern E, Gray J. A mathematical model of the indirect effects of rotavirus vaccination. Epidemiol Infect 2010;138(6):884-97.

14. Steele MK, Remais JV, Gambhir M, Glasser JW, Handel A, Parashar UD, et al. Targeting pediatric versus elderly populations for norovirus vaccines: a model-based analysis of mass vaccination options. Epidemics 2016;17:42-49.

15. Eames KT, Tilston NL, Brooks-Pollock E, Edmunds WJ. Measured dynamic social contact patterns explain the spread of H1N1v influenza. PLoS Comput Biol 2012;8(3):e1002425.

16. Grytdal SP, DeBess E, Lee LE, Blythe D, Ryan P, Biggs C, et al. Incidence of Norovirus and Other Viral Pathogens That Cause Acute Gastroenteritis (AGE) among Kaiser Permanente Member Populations in the United States, 2012-2013. PLoS One 2016;11(4):e0148395.

17. Nordgren J, Svensson L. Genetic Susceptibility to Human Norovirus Infection: An Update. Viruses 2019;11(3).

18. Bureau of Labor Statistics. Occupational employment statistics: May 2018 national occupational employment and wage estimates, United States. 2018 April 2, 2019 [cited 2019 October 9]; Available from: <https://www.bls.gov/oes/current/oes_nat.htm>

19. Centers for Medicare & Medicaid Services (CMS). Physician Fee Schedule. 2018 [cited 2019 2019]; August 15 ]. Available from: <https://www.cms.gov/apps/physician-fee-schedule/>

20. United States Department of Health & Human Services. HCUP facts and figures: statistics on hospital-based care in the United States. 2016 [cited 2019 October 9]; Available from: <http://hcupnet.ahrq.gov/HCUPnet.jsp>

21. Graham DY, Jiang X, Tanaka T, Opekun AR, Madore HP, Estes MK. Norwalk virus infection of volunteers: new insights based on improved assays. J Infect Dis 1994;170(1):34-43.

22. Gray JJ, Cunliffe C, Ball J, Graham DY, Desselberger U, Estes MK. Detection of immunoglobulin M (IgM), IgA, and IgG Norwalk virus-specific antibodies by indirect enzyme-linked immunosorbent assay with baculovirus-expressed Norwalk virus capsid antigen in adult volunteers challenged with Norwalk virus. Journal of Clinical Microbiology 1994;32(12):3059-63.

23. Hall AJ, Rosenthal M, Gregoricus N, Greene SA, Ferguson J, Henao OL, et al. Incidence of acute gastroenteritis and role of norovirus, Georgia, USA, 2004-2005. Emerging Infectious Diseases 2011;17(8):1381-1388.

24. Hall AJ, Curns AT, McDonald LC, Parashar UD, Lopman B. The roles of *Clostridium difficile* and norovirus among gastroenteritis-associated deaths in the United States, 1999-2007. Clinical Infectious Diseases 2012;55(2):216-223.

25. Glass RI, Parashar UE, Estes MK. Norovirus gastroenteritis. New England Journal of Medicine 2009;361(18):1776-1785.

26. Patel MM, Hall AJ, Vinje J, Parashar UD. Noroviruses: a comprehensive review. J Clin Virol 2009;44(1):1-8.

27. Devasia T, Lopman B, Leon J, Handel A. Association of host, agent and environment characteristics and the duration of incubation and symptomatic periods of norovirus gastroenteritis. Epidemiol Infect 2015;143(11):2308-14.

28. Aoki Y, Suto A, Mizuta K, Ahiko T, Osaka K, Matsuzaki Y. Duration of norovirus excretion and the longitudinal course of viral load in norovirus-infected elderly patients. J Hosp Infect 2010;75(1):42-6.

29. Furuya D, Kuribayashi K, Hosono Y, Tsuji N, Furuya M, Miyazaki K, et al. Age, viral copy number, and immunosuppressive therapy affect the duration of norovirus RNA excretion in inpatients diagnosed with norovirus infection. Jpn J Infect Dis 2011;64(2):104-8.

30. Goller JL, Dimitriadis A, Tan A, Kelly H, Marshall JA. Long-term features of norovirus gastroenteritis in the elderly. Journal of Hospital Infection 2004;58:286-291.

31. Teunis PF, Sukhrie FH, Vennema H, Bogerman J, Beersma MF, Koopmans MP. Shedding of norovirus in symptomatic and asymptomatic infections. Epidemiol Infect 2015;143(8):1710-7.

32. Kirkwood CD, Streitberg R. Calicivirus shedding in children after recovery from diarrhoeal disease. J Clin Virol 2008;43(3):346-8.

33. Esposito S, Principi N. Norovirus Vaccine: Priorities for Future Research and Development. Front Immunol 2020;11:1383.

34. Hallowell BD, Parashar UD, Hall AJ. Epidemiologic challenges in norovirus vaccine development. Hum Vaccin Immunother 2019;15(6):1279-1283.

35. Mattison CP, Cardemil CV, Hall AJ. Progress on norovirus vaccine research: public health considerations and future directions. Expert Rev Vaccines 2018;17(9):773-784.

36. Simmons K, Gambhir M, Leon J, Lopman B. Duration of immunity to norovirus gastroenteritits. Emerging Infectious Diseases 2013;19(8):1260-1267.

37. Vanderpas J, Louis J, Reynders M, Mascart G, Vandenberg O. Mathematical model for the control of nosocomial norovirus. Journal of Hospital Infection 2009;71(3):214-22.

38. Heijne JCM, Rondy L, Wallinga J, Kretzschmar M, Low N, Koopmans M, et al. Quantifying transmission of norovirus during an outbreak. Epidemiology 2012;23(2):277-284.

39. Gold MR, Franks P, McCoy KI, Fryback DG. Toward consistency in cost-utility analyses: using national measures to create condition-specific values. Medical Care 1998;36(6):778-92.

40. Rautenberg TA, Zerwes U. The cost utility and budget impact of adjuvant racecadotril for acute diarrhea in children in Thailand. Clinicoecon Outcomes Res 2017;9:411-422.

41. Rautenberg TA, Zerwes U, Foerster D, Aultman R. Evaluating the cost utility of racecadotril for the treatment of acute watery diarrhea in children: the RAWD model. Clinicoecon Outcomes Res 2012;4:109-16.

42. Goossens LM, Standaert B, Hartwig N, Hovels AM, Al MJ. The cost-utility of rotavirus vaccination with Rotarix (RIX4414) in the Netherlands. Vaccine 2008;26(8):1118-27.

43. Yamin D, Atkins KE, Remy V, Galvani AP. Cost-Effectiveness of Rotavirus Vaccination in France-Accounting for Indirect Protection. Value Health 2016;19(6):811-819.

44. Gualano MR, Thomas R, Gili R, Scaioli G, Voglino G, Zotti C. Cost-effectiveness estimates of vaccination against rotavirus in Piedmont, Italy. J Infect Public Health 2018;11(6):867-872.

45. Standaert B, Parez N, Tehard B, Colin X, Detournay B. Cost-effectiveness analysis of vaccination against rotavirus with RIX4414 in France. Appl Health Econ Health Policy 2008;6(4):199-216.

46. Merlo G, Graves N, Brain D, Connelly LB. Economic evaluation of fecal microbiota transplantation for the treatment of recurrent Clostridium difficile infection in Australia. J Gastroenterol Hepatol 2016;31(12):1927-1932.

47. Coyle D, Coyle K, Bettinger JA, Halperin SA, Vaudry W, Scheifele DW, et al. Cost effectiveness of infant vaccination for rotavirus in Canada. Can J Infect Dis Med Microbiol 2012;23(2):71-7.

48. Suwantika AA, Tu HA, Postma MJ. Cost-effectiveness of rotavirus immunization in Indonesia: taking breastfeeding patterns into account. Vaccine 2013;31(32):3300-7.

49. Newall AT, Beutels P, Macartney K, Wood J, MacIntyre CR. The cost-effectiveness of rotavirus vaccination in Australia. Vaccine 2007;25(52):8851-60.
